# Measurement of Centre of Pressure Using the Wii Balance Board in Older Adults with Simulated Visual Impairment

**DOI:** 10.1093/geroni/igab046.2478

**Published:** 2021-12-17

**Authors:** Berkley Petersen, Caitlin Murphy, Aaron Johnson, Karen Li

**Affiliations:** Concordia University, Concordia University, Quebec, Canada

## Abstract

Postural stability is a complex skill dependent upon the coordination of motor, sensory and cognitive systems. The purpose of this project was therefore to explore how older adults’ balance performance is impacted by increased cognitive load, hearing loss, and simulated vision loss. Twenty-eight older adults between the ages of 50 and 93 years (M = 73.86, SD = 10.43) were tested. Participants underwent standard sensory acuity and cognitive functioning tests. The balance trials varied as a function of cognitive load and visual challenge resulting in five conditions: (1) eyes closed, (2) normal vision clear goggles (NV) (3) simulated low vision (20/80) goggles (LV) (4) LV and math task, (5) NV and math task. Postural stability was assessed with three key center of pressure parameters: total path length (TPL), anterior-posterior amplitude (APA) and medial-lateral amplitude (MLA). A mixed-model ANOVA using hearing acuity as a covariate revealed significant effects of complexity in sway amplitude: (APA: p < .017; MLA: p < .020), while TPL approached significance (p < .074). T-tests revealed significant (p < .05) decreases in balance performance across all 3 centre of pressure parameters when comparing single task NV to dual-task NV, NV vs. eyes closed and single task NV vs. LV dual-task. There were significant positive correlations between hearing acuity and balance (MLA) under single task NV (r = .491) and LV conditions (r = .497). Results suggest the attentional demands from increased cognitive load and sensory loss lead to decreases in older adults’ single- and dual-task balance performance.

